# Olfm4 Is Highly Expressed in HCC Patients and as a Biomarker and Therapeutic Target for HCC

**DOI:** 10.1155/2021/5601678

**Published:** 2021-12-06

**Authors:** Yulong Wei, Qingzhu Song, Fenglan Zhang, Tian Yuan

**Affiliations:** ^1^Department of Pathology, Bayannur Hospital, Bayannur, Inner Mongolia 015000, China; ^2^Department of Clinical Laboratory, Bayannur Hospital, Bayannur, Inner Mongolia 015000, China

## Abstract

Hepatocellular carcinoma (HCC) is one of the primary types of cancer that claims many lives worldwide, and its incidence continues to increase. Conventional therapies against liver cancer are inadequate, and the pathogenesis of HCC remains unclear. Thus, not only are more effective therapies to treat HCC required but also identification of the key genes involved in its pathogenesis is important for developing such therapies. This study found that olfactomedin 4 (OLFM4) level is higher in HCC patients than in healthy individuals. Furthermore, HCC patients also have higher messenger ribonucleic acid (mRNA) expression level in HCC tissues than in liver paracancerous tissues. OLFM4 has high predictive capacity as a biomarker for HCC and closely correlates to tumor size. It is confirmed that OLFM4 contributes to cancer cell proliferation, and HIF1*α* is involved in this process. Thus, the OLFM4/HIF-1*α* axis might be a target signaling pathway for developing novel drugs to treat HCC.

## 1. Introduction

Liver cancer is the fourth leading cause of cancer-related deaths after lung, colorectal, and stomach cancer, and its global incidence continues to increase [1]. Hepatocellular carcinoma (HCC) is the most common type of liver cancer, followed by cholangiocarcinoma [2]. The pathogenesis of liver cancer is complicated. Among these, infections, including hepatitis B virus (HBV), hepatitis C virus (HCV), behavioral factors (alcohol and tobacco), metabolic factors (excess body fatness), and aflatoxins are considered major risks [3]. An Italian report indicated that, with the development of new antiviral treatments, early diagnosis approaches of HCC, and improvement of patient surveillance, the epidemiology of HCC has significantly changed in the last decade [4]. In general, chemotherapy and immunotherapy are the most common methods of treating HCC [5]. For the more advanced stages of liver cancer, transarterial chemoembolization (TACE) is often used, which results in 23% improvement in the 2-year survival period compared to conservative therapy for HCC patients in the intermediate stage [6]. As a kinase inhibitor, the oral format of sorafenib is also often used in the latter stages of liver cancer despite its effects being far from satisfactory and long-term drug utilization causing toxicity and/or drug inefficacy [7]. Moreover, the prognosis of liver cancer is low. Consequently, only a small ratio of liver cancer patients is eligible for surgical removal [7]. Therefore, more effective therapies are needed to treat liver cancer. More importantly, identifying the key genes involved in the pathogenesis of liver cancer would help develop novel treatment therapies.

Olfactomedin 4 (OLFM4) belongs to the olfactomedin family, which is also known as the human granulocyte colony-stimulating factor-stimulated clone 1(hGC-1) [8]. Initially, OLFM4 was found to regulate inflammatory response and innate immunity [9]. Moreover, as a secreted protein, OLFM4 is closely involved in a variety of cellular functions, including proliferation, differentiation, apoptosis, and cell adhesion [10]. Recently, evidence confirmed that OLFM4 plays an important role in regulating growth and proliferation of several types of cancer cells [10]. OLFM4 was found to be closely linked to nodal metastases in esophageal adenocarcinoma [11]. The expression level significantly increases in intestinal metaplasia (IM), while it is absent in normal gastric mucosa [12]. In terms of metastatic breast cancer, the expression level of OLFM4 is remarkably associated with the pathological T factor, distant metastasis, and Ki67 status in ER-positive breast carcinoma [13]. OLFM4 is considered a potential biomarker for gastrointestinal cancer [14]. In fact, OLFM4 serum level has been a biomarker for several diseases, including asthmatics [15], non-small-cell lung cancer [16], pancreatic cancer, head and neck cancer, and prostate cancer [17]. However, effects of OLFM4 in liver cancer, including HCC, remain unclear. Therefore, more investigation is needed.

The OLFM4/HIF-1*α* axis was found to be involved in the regulation of hypoxia-induced invasion, epithelial-mesenchymal transition, and chemotherapy resistance in non-small-cell lung cancer [18]. HIF-1*α* is a subunit of a heterodimeric transcription factor hypoxia-inducible factor 1 (HIF-1), which plays an essential role in cellular response to systemic oxygen levels in mammals [18]. HIF-1*α* is closely involved in the pathogenesis of cancer. HIF-1*α* and GATA3 form a complex that enhances cancer cell invasiveness [19]. Targeting HIF-1*α* is a potential therapy for alleviating chemoresistance to enhance the efficacy of chemotherapy in colon cancer [20]. Interestingly, it was found that inhibition of the OLFM4/HIF-1*α* axis could improve hypoxia-induced invasion, epithelial-mesenchymal transition, and chemotherapy resistance of non-small-cell lung cancer [18]. Therefore, in this study, we first measured the OLFM4 level in healthy controls and HCC patients and OLFM4 mRNA and protein level in liver paracarcinoma tissues and tumors. We confirmed that OLFM4 might be a potential biomarker for HCC diagnosis, with high sensitivity and specificity. Finally, we confirmed that silencing OLFM4 could reduce HCC cell proliferation by targeting HIF-1*α*. Thus, this study proposes OLFM4 as a potential biomarker and therapeutic target for HCC and provides critical information for studying pathogenesis and developing novel drugs against HCC.

## 2. Materials and Methods

### 2.1. Patients' Selection

Beginning from May 2016 to May 2020, we recruited 100 HCC patients and 100 healthy controls from the Bayannur Hospital. HCC patients were diagnosed using histopathological analysis. Among 100 HCC patients, 80 received surgery and 20 underwent interventional therapy. HCC was diagnosed according to immunohistochemistry based on the AASLD guidelines. Patients who received radiotherapy or had a history of other solid tumors were excluded. NASH was confirmed based on the histopathology of liver biopsy samples and supported by imaging evidences, such as CT and liver ultrasound. Chronic HBV infection was confirmed by HBsAg presence in the last 6 months with an HBV DNA concentration to >1 × 10^3^ copies per mL, as well as abnormal concentration of serum alanine amino transferase. Confirmation of chronic HCV infection was used for qualitative HCV-RNA measurement, and more than 1 × 103 copies of HCV-RNA in the serum were confirmed to be positive. Healthy controls were identified as without liver or other systematic diseases or HBV markers (HBsAg, HBeAg, anti-HBe, and anti-HBc), as well as normal concentrations of liver function enzymes. The study was approved by the institutional ethics review committee at the Bayannur Hospital. Informed consent was obtained from all participants based on each committee's regulations.

### 2.2. Serum Samples

Serum samples were obtained from patients who were diagnosed with primary HCC at the Bayannur Hospital. Serum from healthy individuals was simultaneously collected at the hospital as control samples. Serum samples were collected under institutional approval. The serum was centrifuged, aliquoted, and stored at −80°C for diagnosis utilization.

### 2.3. Enzyme Linked Immunosorbent Assay

The concentrations of OLFM4 were detected using ELISA. ELISA kits were purchased from Abcam (catalog number: ab267805). ELISA experiments were performed in strict accordance with the manufacturer's instructions.

### 2.4. Reverse Transcription Polymerase Chain Reaction (qRT-PCR)

Total RNA was extracted from HepG2 cells and liver biopsy using Trizol reagent from Invitrogen (Thermo Fisher, catalog number: 15596026). cDNA synthesis was performed using the Maxima Universal First Strand cDNA Synthesis Kit from Thermo Scientific (catalog number: EP0742). RT-qPCR reactions were performed with FastStart Universal SYBR Green Master (Rox) from Roche (catalog number: 04913850001). The experiments were performed according to the manufacturer's instructions.  Table 1 lists the sequence of the primers used for qRT-PCR analyses.

### 2.5. Immunohistochemical Staining (IHC)

For IHC analysis, cancer and paracancerous tissues from liver patients were formalin-fixed, followed by embedding by using the paraffin method. Subsequently, paraffin blocks were prepared into slides, followed by the IHC process using standard instructions. Anti-OLFM4 antibody (ab10586, Abcam1) was used to probe the slides, which were then visualised using DAB + as a chromogen.

### 2.6. Cell Culture and Transfection

Human liver carcinoma cells (HepG2) were purchased from ATCC (ATCC HB-8065). HepG2 cells were maintained in Dulbecco's modified Eagle medium (DMEM, catalog number: 11965118) containing 10% fetal bovine serum (Gibco, catalog number: 10099141C), 100 units/ml penicillin, and 0.1% (w/v) streptomycin (catalog number: 15140163) at 37°C in a humidified atmosphere of 5% CO2.

SiRNAs against OLFM4 and HIF-1*α* against OLFM4 were synthesized by Thermo Fisher Scientific.  Table 2 lists the sequences of siRNAs. The full-length coding sequence of OLFM4 was cloned into pcDNA3.1 vector (Yuanjing Biotechnology, Guangzhou, Guangdong, China). For transfection, siRNAs or plasmids were transfected in HepG2 cells using Lipofectamine 3000 (Thermo Fisher Scientific, catalog number: L3000001) according to the manufacturer's instructions.

### 2.7. Western Blotting

We used the conventional protocol for western blotting (WB). Briefly, cells were lysed for total protein isolation using RIPA lysis buffer (Beyotime, catalog: P0013B). The protein concentration was determined using the Bradford assay. Equal amounts of total protein were separated by SDS-PAGE electrophoresis, transferred to PVDF membranes, and blocked with 5% skim milk powder at room temperature for 1 h. Subsequently, the PVDF membranes were washed with TBST containing NaCl, Tris-HCl, and Tween-20 and incubated with primary antibodies against target proteins, including OLFM4 (Abcam, catalog number: ab267805) and *β*-actin (Abcam, ab8226) at 4°C overnight, followed by two washes with TBST. Thereafter, membranes were incubated with the appropriate secondary antibodies at room temperature for 1 h and washed thrice with TBST. Protein bands were visualised using chemiluminescence (BeyoECL Plus, Beyotime, P0018S, Shanghai, China).

### 2.8. Statistical Analysis

To examine the differences between groups, we used Student's *t*-test and one-way ANOVA. The levels of mRNA expression between cancer tissues and normal tissues were analyzed by the *t*-test. All analyses related to patient survival were tested by Kaplan–Meier survival analysis (log-rank method). A *P* < 0.05 was regarded as statistically significant.

## 3. Results

### 3.1. Patient Characteristics

Table 3 presents the clinical characteristics of the 100 HCC patients, including age, gender, BMI (body mass index), hepatitis infections, NASH status, OLFM4 blood levels, and tumor size. There were 62 females and 58 males; the age (in years) of male patients was 61.87 ± 1.40 and of female patients was 56.75 ± 2.37 (*P*=0.0778). Among female patients, BMI was 20.67 ± 0.27, and it was 20.49 ± 1.86 among male patients (*P*=0.4683). There were 20 HBV-infected female patients and 31 HBV-infected male patients (*P*=0.895); 6 HCV-infected female patients and 7 HCV-infected male patients (*P*=0.668); and 5 NASH female patients and 18 NASH male patients (*P*=0.059). For female patients, the OLFM4 level in blood was 44.06 ± 1.67 (U/L), and it was 43.16 ± 2.23 (U/L) (*P*=0.7815) for male patients. For female patients, the tumor size was 2.683 ± 0.30 (mm, diameter), and it was 43.16 ± 2.23 (mm, diameter) (*P*=1.72 ± 0.14) for male patients.

### 3.2. OLFM4 Expression in Blood and Tissues

**Figure 1:**
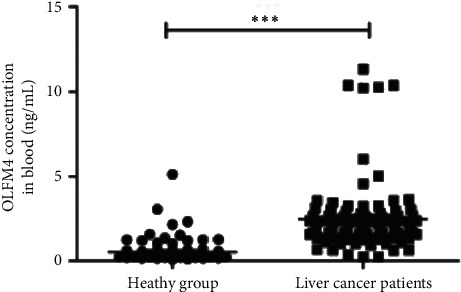
The blood level of OLFM4 in healthy individuals and HCC patients.  ^*∗∗∗*^*P* < 0.001.

**Figure 2:**
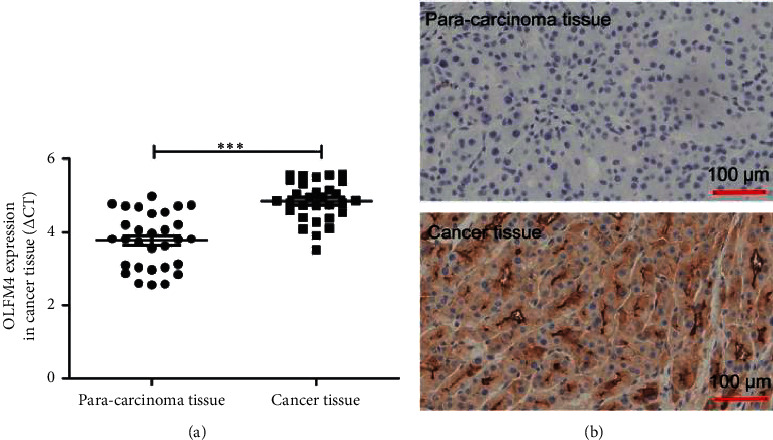
mRNA and protein levels of OLFM4 in paracarcinoma tissue and cancer tissue in HCC patients. (a) mRNA expression of OLFM4 in paracarcinoma tissue and cancer tissue in HCC patients measured by qRT-PCR assay,  ^*∗∗∗*^*P* < 0.001; (b) protein levels of OLFM4 in paracarcinoma tissue and cancer tissue in HCC patients measured by IHC assay.

To test the potential of OLFM4 as a biomarker of HCC, OLFM4 blood level was measured using ELISA. OLFM4 level in HCC patients was significantly higher than in healthy individuals (Figure 1, *P* < 0.001). To further demonstrate, the mRNA expression level of OLFM4 in liver paracancerous and cancer tissues was measured. The mRNA expression level of OLFM4 in HCC tissues was remarkably higher than that in liver paracancerous tissues (Figure 2(a), *P* < 0.001). OLFM4 staining was observed in HCC tissues but not in liver paracancerous tissues (Figure 2(b), *P* < 0.001). Thus, OLFM4 expression level was significantly upregulated in HCC patients.

### 3.3. Diagnostic Capability of OLFM4 Expression and Correlation with Tumor Size

**Figure 3:**
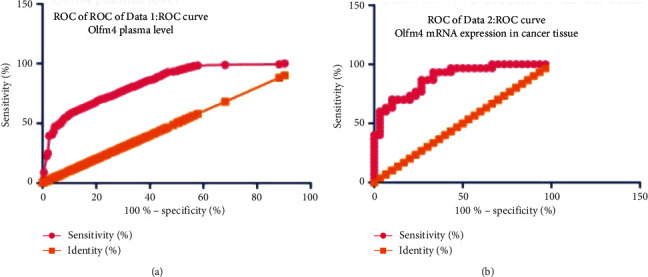
ROC analysis of the performance of OLFM4 expression in identification of HCC. (a) ROC analysis using the blood level of OLFM4 to predict HCC; (b) ROC analysis using the mRNA expression of OLFM4 in liver cancer tissue to predict HCC.

Receiver operating characteristic (ROC) analysis was performed to determine the diagnostic value of OLFM4 expression for HCC (Figure 3). It indicated that both OLFM4 level (Figure 3(a)) and mRNA expression level in liver tissues (Figure 3(b)) had excellent diagnostic value overall; the AUCs were 0.9292 (*P* < 0.0001) and 0.8844 (*P* < 0.0001), respectively.

**Figure 4:**
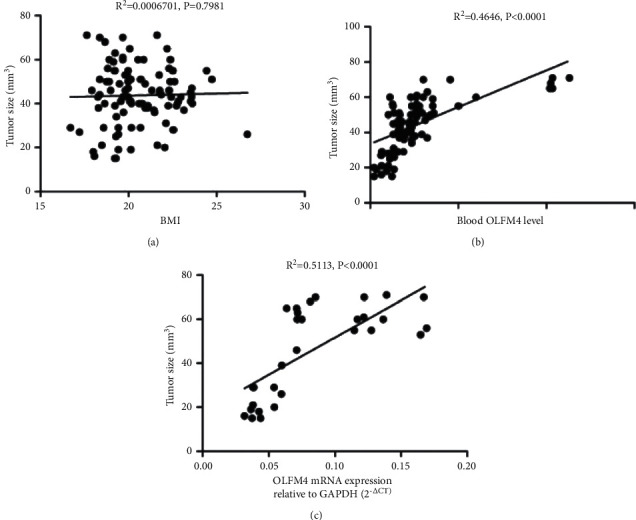
Correlation of OLFM4 to tumor size. (a) Correlation analysis about BMI to tumor size; (b) correlation analysis about OLFM4 blood level to tumor size; and (c) correlation analysis about mRNA expression of OLFM4 in HCC tissue to tumor size.

To further assess the diagnostic value of OLFM4 for HCC, the correlation between OLFM4 expression and tumor size was analyzed. As shown in Figure 4, BMI had no clear correlation to tumor size (Figure 4(a)). Interestingly, OLFM4 level has a significant correlation to tumor size (*R*^2^ = 0.4646, *P* < 0.0001) (Figure 4(b)). OLFM4 mRNA expression in HCC tissue was significantly correlated to tumor size (*R*^2^ = 0.5113, *P* < 0.0001) (Figure 4(c)). Collectively, OLFM4 has a diagnostic value to predict HCC, and it significantly correlated to tumor size.

### 3.4. OLF4M Closely Regulated Proliferation of HepG2 Cells

**Figure 5:**
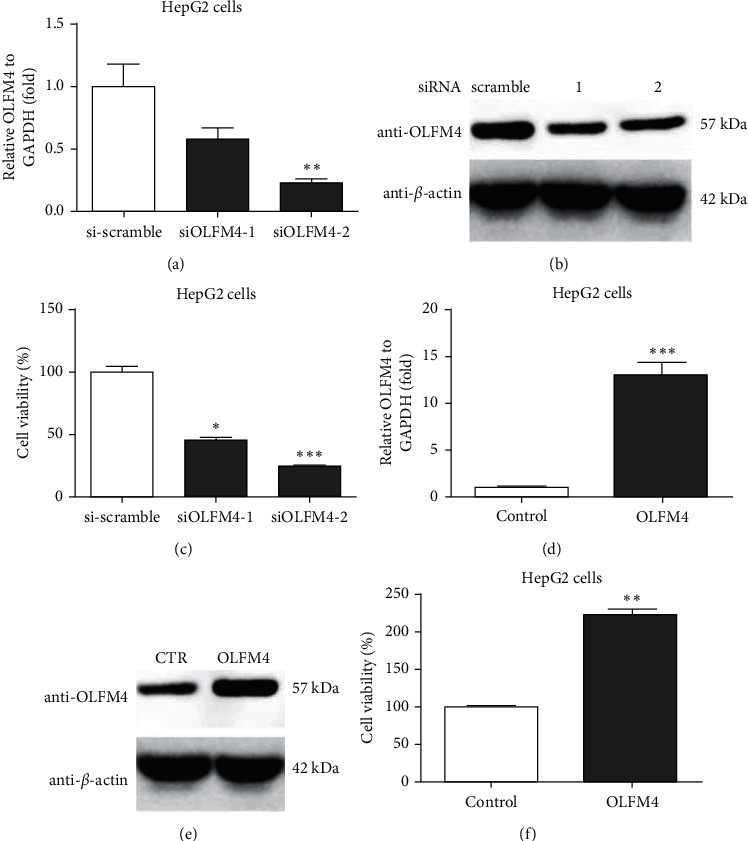
OLFM4 regulated liver cancer cell proliferation in HepG2 cells. (a) siRNAs against OLFM4 successfully inhibited the mRNA expression of OLFM4 in HepG2 cells measured by qRT-PCR assay ( ^*∗∗*^*P* < 0.01); (b) siRNAs against OLFM4 successfully inhibited the protein level of OLFM4 in HepG2 cells measured by western blot assay; (c) siRNAs against OLFM4 could inhibit the proliferation of HepG2 cells ( ^*∗*^*P* < 0.05,  ^*∗∗*^*P* < 0.01); (d) OLFM4 plasmid transfection could overexpress the gene in HepG2 cells detected by qRT-PCR assay ( ^*∗∗∗*^*P* < 0.001); (e) OLFM4 plasmid transfection could upregulate the protein level of OLFM4 in HepG2 cells detected by western blot assay; and (f) OLFM4 plasmid could affiliate the proliferation of HepG2 cells ( ^*∗∗*^*P* < 0.01).

To investigate the effects of OLFM4 on HCC, a human liver carcinoma cell line (e.g., HepG2) was used. First, two siRNAs against OLFM4 were constructed, and both siRNAs showed knockdown effects on OLFM4 in HepG2 cells, while the second siRNA showed better knockdown efficiency (Figure 5(a)). Knockdown was further verified by WB assay (Figure 5(b)). Effects of siRNAs against OLFM4 on HepG2 proliferation were measured using CCK8 assay, which indicated that two siRNAs significantly reduced HepG2 proliferation (Figure 5(c)). Simultaneously, OLFM4 overexpression plasmids were constructed, which indicated significant regulation of the OLFM4 gene in HepG2 cells after transfection (Figure 5(d)). WB assay confirmed overexpression in HepG2 cells (Figure 5(e)). Moreover, OLFM4 overexpression significantly promoted cell proliferation in HepG2 cells (Figure 5(f)). Collectively, it was demonstrated that OLFM4 closely regulates HCC cell proliferation.

### 3.5. HIF-1*α* Involved in the Regulation of OLFM4 on HCC

**Figure 6:**
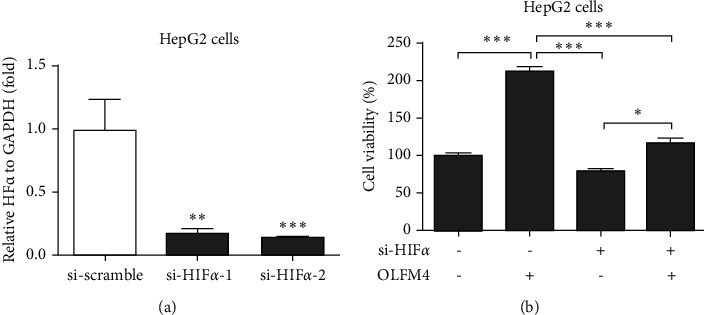
HIF1*α* is involved in the regulation of OLFM4 in liver cancer proliferation. (a) siRNAs against HIF1*α* successfully inhibited the mRNA expression of HIF1*α* in HepG2 cells measured by qRT-PCR assay ( ^*∗∗*^*P* < 0.01,  ^*∗∗∗*^*P* < 0.001); (b) the knockdown of HIF1*α* alleviated the increase of OLFM4 plasmid on liver cancer proliferation in HepG2 cells.

HIF-1*α* was found to be the downstream gene of OLFM4, which encouraged us to study whether HIF-1*α* is involved in the regulation of OLFM4 in HCC. Two siRNAs against HIF-1*α* were synthesized and transfected in HepG2 cells, which indicated a significant knockdown (Figure 6(a)). It was found that the knockdown of HIF-1*α* significantly alleviated the promotion of OLFM4 on HepG2 proliferation (Figure 6(b)). Thus, we confirmed that HIF-1*α* involves the regulation of OLFM4 on HCC cell proliferation.

## 4. Discussion

HCC has been become one of most severe types of cancer claiming many lives worldwide [21]. Although several therapies, including chemotherapy, arterial embolization, surgical resection, and radiofrequency ablation, have been developed to treat the disease, none of them are ideal due to native side effects [3]. Furthermore, the pathogenesis of HCC is still unclear. Noninvasive diagnosis for HCC is needed because conventional methods, such as liver biopsy, may cause significant morbidity [22]. In the present study, we first measured OLFM4 level in HCC patients and healthy controls and mRNA expression level in liver paracancerous and cancer tissues. The HCC patients had higher OLFM4 level, and HCC tissues had higher OLFM4 expression level than liver paracancerous tissues. ROC analysis indicated that OLFM4 had a high diagnostic value for HCC. OLFM4 had a strong correlation with tumor size. Finally, we confirmed that OLFM4 contributed to HCC cell proliferation in HepG2 cells, and HIF-1*α* is involved in the regulation of OLFM4 on HCC cell proliferation.

Cancer incidence varies greatly depending on gender. For example, it was found that gender differences existed in cancer-associated venous thromboembolism [23]. Yang et al. found that men and women had different colorectal cancer survival [24]. In case of lung cancer, it was found that long-time survival after curative resection in early stage, non-small-cell lung cancer is better in women than in men, and women often showed more molecular changes than men [25]. However, in our study, gender differences were found for OLFM4 level and tumor size in HCC patients (Table 3). Whether HCC entails gender-based differences needs further discussion since our sample was limited.

HCC has been found to induce the expression modification of a large body of genes. Zhang et al. used bioinformatics analysis to identify several key genes and pathways in HCC, including GMPS, ACACA, ALB, TGFB1, KRAS, ERBB2, BCL2, EGFR, STAT3, and CD8A [26]. Similarly, Shen et al. also found the expression level of a panel of genes, such as TOP2A, NDC80, FOXM1, HMMR, KNTC1, PTTG1, FEN1, RFC4, SMC4, and PRC1, significantly changed in HCC [27]. These genes might be potential noninvasive biomarkers for the diagnosis of HCC. Pan et al. found that SLC25A11 was downregulated in HCC compared to normal controls, and low expression of SLC25A11 was significantly associated with the clinical stage, vital status, histologic grade, overall survival (OS), and relapse-free survival (RFS). Thus, SLC25A11 may serve as a prognostic marker for liver cancer [28]. OLFM4 is generally considered as a marker of stem cells. Interestingly, Suzuki et al. found that OLFM4 expression was associated with nodal metastases in esophageal adenocarcinoma, and it might be an informative marker with the potential to improve preoperative assessment in patients with esophageal adenocarcinoma [11]. van der Flier et al. found that OLFM4 was a robust marker for stem cells in the human intestine, and it marks a subset of colorectal cancer cells [29]. Mayama et al. found that OLFM4, LY6D, and S100A7 could be potent markers for distant metastasis in estrogen receptor-positive breast carcinoma [30]. Consistently, we found that OLFM4 level was higher in HCC patients compared to the healthy controls, and mRNA expression was higher in HCC tissues than that in liver paracancerous tissues (Figures 1 and 2). We also found that OLFM4 has a high predictive value for diagnosing HCC and is closely correlated to tumor size (Figures 3 and 4). However, Clemmensen et al. analyzed the OLFM4 plasma level in the healthy controls and patients with gastrointestinal cancer; there was no association between OLFM4 plasma level and colorectal malignancy [14]. Therefore, OLFM4 might be a potential noninvasive biomarker for several cancer types, including HCC. However, more clinical and experimental data should be accumulated to further verify the predictive capacity in terms of HCC.

Seeking a therapeutic target is vital for developing effective drugs to treat cancer. This study found that OLFM4 promoted HCC cell proliferation (Figure 5). In gastric cancer cells, it was found that depletion of the OLFM4 gene inhibited cell growth and increased sensitization to hydrogen peroxide, and TNF*α* induced apoptosis [31]. Consistent with our results, Ashizawa et al. reported that OLFM4 could activate STAT3 and affiliate tumor progression by decreasing the expression level of GRIM19 in human HCC [32]. Interestingly, Gao et al. demonstrated that HIF-1*α* is involved in the regulation of OLFM4 on hypoxia-induced invasion, epithelial-mesenchymal transition, and chemotherapy resistance in non-small-cell lung cancer [18]. This study also confirmed that HIF-1*α* is involved in the regulation of OLFM4 in terms of HCC cell proliferation (Figure 6). Thus, the OLFM4/HIF-1*α* axis might be a target signaling pathway for developing novel drugs to treat HCC.

In summary, OLFM4 level is higher in HCC patients than in healthy individuals, and mRNA expression level is higher in HCC tissues than in liver paracancerous tissues. OLFM4 has high predictive capacity as a biomarker for HCC and is closely correlated to tumor size. Most importantly, we confirmed that OLFM4 contributes to cancer cell proliferation, and HIF-1*α* is involved in this process. We believe that the OLFM4/HIF-1*α* axis might be a target signaling pathway for developing novel drugs to treat HCC.

## 5. Conclusions

The main findings and implications of the work are clearly explained, highlighting its importance and relevance.

## Figures and Tables

**Table 1:** Primers of OLFM4 and HIF1*α*.

Gene	Primer type	Sequence	Tm	Product size
OLFM4	Sense	TCAGCAAACCGTCTGTGGTT	60.11	70
	Antisense	TCCCTACCCCAAGCACCATA	59.95	

HIF1*α*	Sense	GTCTGAGGGGACAGGAGGAT	60.03	80
	Antisense	CTCCTCAGGTGGCTTGTCAG	60.04	

**Table 2:** Sequences of siRNAs.

Genes	siRNA	Sequence	GC%
OLFM4	OLFM4#1	AAGACCAAGCTGAAAGAGTGT	42.86
	OLFM4#1	AAGGATACCATTTCTTACACT	33.33

HIF1*α*	HIF1*α*#1	AAGGATGCAAATCTAGTGAAC	38.10
	HIF1*α*#2	AAGGACAAGTCACCACAGGAC	52.38

**Table 3:** Basic physiological characteristics of HCC.

		Female	Male	*P* value
Patients (*n*)		38	62	
Age (years; means ± SEM)		61.87 ± 1.40	56.75 ± 2.37	0.0778
BMI		20.67 ± 0.27	20.49 ± 1.86	0.4683
Hepatitis infections	HBV+	20	31	0.895
	HCV+	6	17	0.668
NASH		5	18	0.059
OLFM4 blood levels (U/L) (mean ± SEM)		44.06 ± 1.67	43.16 ± 2.23	0.7815
Tumor size (mm, diameter) (means ± SEM)		2.683 ± 0.30	1.72 ± 0.14	0.7760

## Data Availability

Some or all data, models, or code generated or used during the study are available from the corresponding author on request.
